# Haemoglobinopathies in Europe: health & migration policy perspectives

**DOI:** 10.1186/1750-1172-9-97

**Published:** 2014-07-01

**Authors:** Patricia Aguilar Martinez, Michael Angastiniotis, Androulla Eleftheriou, Beatrice Gulbis, Maria Del Mar Mañú Pereira, Roumyana Petrova-Benedict, Joan-Lluis Vives Corrons

**Affiliations:** 1 CHU de Montpellier, Saint Eloi Hôpital, Montpellier, France; 2 Thalassaemia International Federation, Nicosia, Cyprus; 3 Clinical Chemistry Department, Hôpital Université Libre de Bruxelles, Brussels, Belgium; 4 Red Cell Pathology Unit, Hospital Clinic. University of Barcelona, c/ Villarroel 170, 08036 Barcelona, Spain; 5 International Organization for Migration (IOM), Migration Health Division (MHD), Regional office (RO), Brussels, Belgium

**Keywords:** Haemoglobinopathies, Thalassaemia, Sickle cell disease, Population migration, Migrant health, Europe, Policy recommendations

## Abstract

**Background:**

Major haemoglobinopathies (MH), such as thalassaemia syndromes (Thal) and sickle cell disorders (SCD), are genetic defects associated with chronic anaemia and other complications. In Europe, MH are rare diseases (RD) but their prevalence is significantly growing in many countries due to mobility and migration flows. This creates a growing health problem in the EU that has not yet been effectively addressed by Member States (MS) authorities. The present study has been conducted with the aim of: (i) providing an overview of policies for MH in 10 EU member states (MS) (ii) analysing the challenges linked to these RD due to growing requirements imposed by population, mobility and migration trends and (iii) identifying gaps, proposing improvements on existing policies, or developing new ones to fit the identified needs.

**Methods:**

The study has been undertaken by a group of members of the European Network for Rare and Congenital Anaemias (ENERCA) and the Thalassaemia International Federation (TIF), in collaboration with the public affairs firm Burson-Marsteller Brussels. Data from 10 EU countries have been gathered using targeted desk research and one-to-one interviews with local stakeholders, including healthcare professionals, patients and public health officers/providers.

**Results:**

1. MH are the most common RD in all the 10 countries, 2. Data on prevalence, overall burden, trends, and clinical follow up costs are lacking in most countries. 3. Neonatal screening practices show a wide variation across and within countries. 4. Awareness on MH and their related complications is very low, exception made of Italy, Greece, Cyprus and UK, 5. No disaggregated data is available to understand the impact of mobility and migration on the prevalence of haemoglobinopathies, and how healthcare delivery systems should adapt to respond to this situation. 6. Targeted policy measures and/or actions are generally lacking and/or delayed.

**Conclusions:**

Ten policy recommendations have been drawn from this study, building on 2006 WHO recommendations for MH to include haemoglobinopathies in National Plans of Actions for Rare Diseases.

## Background

Major Haemoglobinopathies (MH), mainly thalassaemia syndromes (Thal) and sickle cell disease (SCD) are rare genetic blood disorders that affect more than 330,000 newborns every year worldwide. From these, about 70% suffer from SCD, and the remaining, from severe forms of Thal [1]. The World Health Organization (WHO) has stressed that haemoglobinopathies are a growing health problem in 71% of 229 countries, which account for 89% of all births worldwide, mostly in low and middle income countries [2]. Their prevalence, however, varies considerably between the different regions of the world, between countries in the same region, between areas within a country, and even, between different medical centres within the same area (2). These disorders are endemic in the Mediterranean, African and Asian regions; and are currently the most common rare diseases (RD) of genetic origin in Europe [3,4].

In addition to severe anaemia, both Thal and SCD, can lead to chronic complications that severely undermine the quality of life of patients or lead to an early death (Table 1). If untreated, patients with thalassaemia major usually die during childhood or before adolescence. Transfusion therapy has resulted in increased life expectancy; however, patients develop iron overload and related complications in the cardiac, hepatic and endocrine system. In addition, patients may develop infections, mainly transfusion related (e.g. Hepatitis B and C), bone abnormalities and osteoporosis. In SCD, patients suffer from chronic or acute anaemia due to increased haemolysis, but also from adverse events related to hyperviscosity and vaso-occlusion. Clinical manifestations include acute pain (vaso-occlusive crisis), hyposplenism due to multiple splenic microinfarcts, acute chest syndrome, osteonecrosis, nephropathy, proliferative retinopathy, leg ulcers, severe vasculopathy and cerebrovascular disease. Patients with SCD are similarly at increased risk of infections related to hyposplenism [5-7].

**Table 1:** Co-morbidities and/or complications caused by major haemoglobin disorders

**Thalassaemia major and intermedia**	**Sickle-cell disease**
Chronic Anaemia (if untreated)	Chronic anaemia
	Acute anaemia (increase haemolysis, bone marrow suppression, blood loss, spleen sequestration, malarial infection, etc.)
Iron overload (from the disease or blood transfusion) and its complications in the cardiac, hepatic and endocrine system	Adverse events related to hyperviscosity and vaso-occlusive crisis (Acute pain, acute chest syndrome, hyposplenism, nephropathy, proliferative retinopathy, etc.) and haemolysis and vasculopathy (Pulmonary hypertension; priapism, leg ulcers and cerebrovascular disease)
Increased risk of infections (mainly transfusion related e.g. Hepatitis B and C)	Increased risk of infections related to hyposplenism (And to transfusions in transfused patients)
Bone abnormalities and osteoporosis	Risk of osteonecrosis

Through mobility and migration flows, haemoglobinopathies have spread from the Mediterranean, Africa and Asia to the whole Europe, the Americas and Australia, and there is scientific evidence that they have become a global public health problem. Because they are endemic or have expanded following mobility flows, haemoglobinopathies are present in all European countries creating an important impact on health services [4]. However, there are still poor data on the precise prevalence, overall burden, and trends of the diseases, due to a lack of comprehensive data collection and analysis systems in the different European countries.

As the consequence of medical advances in the last decades, haemoglobinopathies can be effectively prevented, early and specifically diagnosed, and treated appropriately, as a mean to prevent secondary complications and increase patients’ life expectancy or quality of life [8,9]. Accordingly, in countries with existing standards of care and where management guidelines have been implemented and well adhered to by patients, the rate of survival and quality of life has significantly increased.

Prevention and management of haemoglobin disorders is well established and managed in countries where these conditions were traditionally endemic (e.g. Cyprus, Greece, Italy) or in countries that have a longstanding tradition of receiving migrants (e.g., France and the UK). However, current and future mobility and migration flows to and within regions including the European Union (EU), pose considerable new challenges that have to be taken into consideration by members states (MS) and EU authorities. In a number of Europe countries, with “low incidence rates” of haemoglobinopathies, the evidence shows that migrant population groups (e.g. populations from Southern Europe, North Africa, Southern Asia and Southeast Asia) are under-diagnosed, and particular care needs are not sufficiently recognised and embedded into healthcare delivery systems [10-13].

The aim of this study was to analyse and evaluate current health and social policies affecting patients with MH in 10 European countries, to identify main policy gaps and best practices, and, based on these, to suggest potential actions. Ten essential recommendations for policy actions have been put forward for consideration by the EU, national governments, health authorities and providers.

## Methods

The study focused on the following EU Member States (MS): Belgium, Cyprus, France, Germany, Greece, Italy, the Netherlands, Spain, Sweden and the UK. It was undertaken, between March and October 2012, by a group of members of the European Network on Rare and Congenital Anaemias (ENERCA), specialized in the field of haemoglobinopathies, and the Thalassaemia International Federation (TIF), in collaboration with Burson-Marsteller Brussels, a public affairs and communication agency.

Through desk research and performing interviews using a structured questionnaire (Additional file 1 relevant information was gathered from each of the 10 MS with regard to the prevalence of Thal and SCD, existing guidelines and practices at national or regional level, relevant policies, and the healthcare cost of these disorders. Information came from a range of sources including the local governments, patient’s associations, policy documents, scientific literature and media reports. Building on the desk research undertaken, one-to-one interviews were carried out in each of the ten countries, with three to four national stakeholders from relevant government services, physicians and other healthcare professionals, patients and families. While attempts were made to consult a range of stakeholder groups in the preparation of each country report, exceptionally responses from all groups, including government officials, were not available within the given time period in all countries. In such situations, the reports were written on the basis of the best information available through the desk research and other interviews.

On the basis of the gathered information, individual country reports were built. The information was structured as follows: (i) Key facts and figures, (ii) Policy focus, (iii) Prevention and screening, (iv) Treatment and care, (v) Policy outlook and (vi) Interviewee’s suggested policy actions.

## Results and discussion

According to the World Health Organization (WHO), approximately 5% of adults, in the word, are carriers for a haemoglobin disorder, 2.9% for thalassaemia and 2.1% for SCD [1]. Moreover, over 300,000 children are born each year with a MH, 30% are born with Thal, while 70% have SCD. With worldwide migration, these diseases are as much a feature of Europe, the United States and Australia as of the countries where they originated, representing a growing health problem in 71% of 229 countries, which account for 89% of all births.

### Prevalence and burden of haemoglobinopathies and the impact of migrations

The number of patients with haemoglobinopathies has been estimated to be almost 44,000 in the 10 EU countries analysed in this survey, varying from 150 cases in Sweden to 10,500 in France.^.^ Recent migration flows to and within the EU have increased the number of patients with haemoglobinopathies in many European countries [3,4]. However, accurate, comprehensive and disaggregated data is missing in the vast majority of the countries in order to understand how mobility and migrant flows are impacting haemoglobinopathies epidemiology and how healthcare systems and policies are responding to this. Even where data collection and analysis systems do exist, these do not address relevant migration and ethnic origins of patients comprehensively through relevant disaggregated data, as recommended by WHO [14,15]. Moreover, there are no direct cost estimates available on the financial and social burden of haemoglobin disorders as compared to the long-term costs associated to misdiagnosis and poor management of the diseases. However lifetime treatment costs have been estimated by health economists [16], which do confirm a rising cost as patients grow older. The cost–effectiveness of prevention has been studied more deeply [17,18].

### Policy focus

There is a considerable absence of targeted and comprehensive policy measures across the 10 EU countries included in this report, with some notable exceptions (Table 2). In 2006, WHO, published two Resolutions addressing specifically the health needs on SCD and Thal [19,20]. Despite this, the establishment of comprehensive plans to address Thal and SCD is lacking in most of the studied European MS, with the exception of five countries, of which three are countries where haemoglobin disorders are endemic (Cyprus, Greece, Italy and France and UK). These countries have specific strategies or programmes in place dedicated to haemoglobinopathies. These include: the prevention and holistic management of the diseases, screening, education and awareness measures and aim to take into account cultural practices to a certain extent. Accordingly, in only two of the studied EU countries where these disorders are mainly imported via migrations, they remain a secondary priority for policy action and, in countries where actions do exist, these are fragmented. Moreover, there is no particular reference or focus within RD strategies or migrant health initiatives on haemoglobin disorders. Reluctance or delay from governments to take targeted action to address MH effectively may be partly attributed, depending on the countries, to the underestimation of the burden of these diseases and of the costs associated with poor or inadequate screening, diagnosis and clinical management. More recently, cost-containment measures in healthcare budgets, as well as outstanding ethical, legal and social concerns linked to screening and prevention of genetic disorders, have further contributed to the governments’ reluctance to take action. In addition, structured dialogue between governments and the relevant healthcare communities or patient’s associations is very limited or, in most countries, inexistent.

**Table 2:** Haemoglobinopathies: summary indicators in 10 European countries

**Haemoglobinopathies report summary of indicators**									
**Country**	**Perceived influence of migration on epidemiology**	**National/ local THAL registry**	**National/local SCD Registry**	**Structured national neonatal screening programme**	**Structured national antenatal screening programme**	**National/regional rare disease plan expected by**	**Haemoglobin disorders focused plans or focus in rare disease plans**	**Reimbursement of treatments**	**Country awareness**
**Belgium**	high influence	no	national	local	No rules	yes, work in progress	no	yes	low
**Cyprus**	low influence traditionally, but increasing	national	no	no	Yes, by church and civil authorities (for the Turkish community)	yes approval planned in 2013	yes	yes	very good
**France**	high influence	national	local	National, targeted to the at risk population	Antenatal diagnosis available upon request	Yes (already implementd)	yes	partly	low
**Germany**	high influence	no	no	no (trial Berlin)	no	yes, work in progress 2013	N/A	partly	very low
**Greece**	low influence: disorders linked to indigenous to population	National (on Haemoglobinopathies)		yes	yes	yes	yes	yes (for insured patients and asylum seekers)	high to some extent only
**Italy**	low influence: disorders linked to indigenous to population	regional (on Haemoglobinopathies)		local/regional	local/regional	yes, work in progress 2013	N/A	yes (depending on the region)	generally low, high in specific regions
**The Netherlands**	high influence	local	local	yes	yes	yes, work in progress 2013	N/A	yes	low
**Spain**	high influence	National scientific registry not official	National paediatric scientific registry	regional	No rules	yes	no	In general yes, some regions partially	low
**Sweden**	high influence	no	no	no	no	yes, work in progress 2013	N/A	yes	very low
**UK**	high influence	yes	yes	yes	yes	yes, work in progress 2013	yes	yes	relatively high

### Prevention and diagnosis

Screening practices vary across and within the countries surveyed, and it was found that they generally target the population considered at risk, or being carriers of the disease (Table 2). In most cases, screening is not structured and comprehensively implemented within countries and is dependent on the knowledge and education of both healthcare professionals and patients. At risk population is generally defined by ancestors’ origin or ethnicity, based on physical appearance or presumption from healthcare professionals. In only limited cases exceptionally there are clear guidelines to define screening target population. In the UK, experiments of screening based on historical environmental exposure and a targeted questionnaire has been established in order to improve screening coverage [21]. Haemoglobinopathies are usually not covered in medical undergraduate curricula, and awareness of the risks of being a carrier, affected birth rates, associated health risks and sequels and how to prevent and manage haemoglobin disorders, were low among the general population. Language barriers and poor multicultural competencies of healthcare staff and support teams were also considered critical factors leading to under or misdiagnosis, poor disease management and patient adherence with treatment, lower patient safety and patient awareness. Only a few countries, including Cyprus, Greece, Italy, Belgium and the UK, have national or local targeted education and awareness campaigns to prevent haemoglobin disorders amongst the general population. Such campaigns were mostly driven by patient support organisations in France, the Netherlands, Spain and the UK. In Cyprus, Greece and Italy, there have been successful prevention experiences by raising general awareness with the involvement of the broader community (educational and religious bodies) [9,22].

### Treatment and care

Based on the retrieved country policies or on the interviews performed in each country with governments, health professionals or patient support groups, it appears that patients with haemoglobin disorders are generally treated by specialist doctors in hospitals or dedicated centres. Timely, regular access to specialist care, innovative monitoring methods for diagnosis and treatment outcomes varies across and within countries with healthcare cuts and specialist shortages being the main reasons behind the variations. Specialist care and follow-up of medical complications primary or secondary to the disease in the context of a multi disciplinary approach are considered an essential component in the effective management of the haemoglobinopathies in order to reduce mortality and health complications. However, due to a multidisciplinary requirement, the approach to the care of these patients is generally fragmented and coordination of specialists from different relevant disciplines is often not structured. Specialist medical associations or patients’ organizations like Thalassaemia International Federation usually drive the preparation and publication of guidelines and protocols. However, awareness of health professionals and effective implementation across the countries is inconsistent. The recognition of European Reference Networks in rare diseases [23] may strongly facilitate the wide diffusion of good practices and up to date standards of care. An example is the European Network for Rare and Congenital Anaemias (ENERCA) that has recently published guidelines for the management of SCD in adults and children [13] and is currently working on the establishment of a European network of centres of expertise on Rare Anaemias in Europe [24].

### Social support

Interviews have also stressed the fact that psychological and professional support to help patients improve their quality of life and integrate into the labour market is scarce, fragmented and often undertaken by the patients support group; and that it is often forgotten in the management of these diseases. Financial support to affected patients is habitually provided through general support measures for people with disabilities, under national rules, but additional costs linked to care (e.g. transport to hospitals for regular treatment) are often not covered.

## Conclusions

### Policy recommendations

On the light of the results here obtained, 10 essential policy recommendations can be drawn. Target groups, means of implementation and potential obstacles for each recommendation are summarized in Table 3:

**Table 3:** Ten major recommendations to improve diagnosis and care of patients with haemoglobin disorders in European countries

**Recommendation**	**Target**	**Means of implementation**	**Potential obstacles to be overcome**
1. Develop and effectively implement data collection and analysis systems	Health professionals, Ministry of health, Epidemiologists	Establish registries and databases to ensure adequate and consistent collection and analysis of data	Financial, Ethical and legal
2. Develop and implement targeted programmes to address haemoglobinopathies	Heath professional, Specialized physicians, Primary care doctors (for the initial diagnosis and every day follow up), Healthcare managers	Develop medical education, professional guidance and protocols on prevention, management and treatment of Hb disorders, Improve access to innovative screening such as MRI or intracranial echo doppler	Financial
3. According to the specific national needs, provide for adequate specific measures to address haemoglobin disorders in the framework of National Rare Disease Plans	National health authorities, Governments	Comprehensive action plan with targeted actions and measurable objectives; shaped in dialogue with the healthcare community and patients	Poor awareness of broader societal burden of the diseases and impact assessment of targeted plans
4. Establish centres of reference or expertise for: Harmonized and easy access to diagnosis, treatment and specialised care and comprehensive follow-up of patients, Preventing and improving early and specific diagnosis of haemoglobin disorders by the development of newborn screening programmes for sickle cell disease where necessary and their protocols to ensure consistent screening practices,	National health authorities, Governments, National health authorities, Governments, Healthcare professionals and managers	Adoption of ENERCA recommendation for recognition of Centers of Expertise at National level	Lack or delayed implementation of National Plans or Strategies for rare diseases.
5. Fund research on haemoglobinopathies	International, EU and national authorities in charge of research programmes, Pharmaceutical companies	Rare Anaemias have to be included in the European and National Research Programmes	Financial restrictions
6. Implement and support education and awareness measures targeted at the general public, haemoglobin patients and potential carriers, including different migrant and ethnic minority groups	General public, Patients, Patient support groups, Health professionals, National Health Authorities	Effective information and education measures should be tailored to the different range of ethnic minority groups. Easily accessible information materials should be developed, which target couples and children and describe the most important aspects of haemoglobinopathies diagnosis and prevention.	Financial, Language and cultural barriers
7. Implement and actively support targeted healthcare professional education and training programmes with a particular focus on the prevention, diagnosis and management of haemoglobinopathies	European and national Scientific Societies, Ministries of Health and ministries of education	Increase the part given to rare anaemias in the European Haematology curricula and continuing medical education	Lack of multicultural care competencies development of healthcare practitioners, Lack of dedicated health professionals
8. Support the empowerment and participation of patients and the healthcare community	Patients Associations and Health care Community	Increase of communication channels between patients and professionals and shape policies that respond to the specific needs of patients with haemoglobinopathies and develop multicultural healthcare delivery methods	Social and cultural inequalities across Europe
9. Support the development and implementation of guidelines and standards of care and prevention of haemoglobin disorders and linked sequels	Scientific and professional societies and National health care Authorities	Development of coordinated activities between health professionals and authorities for improving the quality of healthcare delivery	The existence of inequalities in the health care provision across Europe and within individual countries
10. Adopt specific measures aimed at addressing healthcare staff shortages	Health Authorities and Medical Centres	Diversification of health staff origins, increasing the professional/patients ratio and assuring multidisciplinary care	Lack of specialists and financial restrictions

1. Develop and effectively implement data collection and analysis systems

2. Develop and implement targeted programmes to address haemoglobinopathies

3. According to the specific national needs, provide for adequate specific measures to address haemoglobin disorders within the framework of National Rare Disease Plans

4. Establish centres of reference or expertise according to the recommendations for expert centres in Rare Anaemias recognition published in ENERCA WHITE BOOK [24]

5. Fund research on haemoglobinopathies

6. Implement and support education and awareness measures targeted at the general public, haemoglobin patients and potential carriers, including different migrant and ethnic minority groups. Awareness is particularly important in the promotion of effective prevention and this should include professionals in primary health care, as well as the general public. A striking example of the importance of awareness is the need to screen for these disorders in early pregnancy so that prenatal diagnosis may be a possible choice.

7. Implement and actively support targeted healthcare professional education and training programmes with a particular focus on the prevention, diagnosis and management of haemoglobinopathies

8. Support the empowerment and participation of patients and the healthcare community

9. Support the development and implementation of guidelines and standards of care and prevention of haemoglobindisorders and linked sequels

10. Adopt specific measures aimed at addressing healthcare staff shortages

## Abbreviations

SCD: Sickle cell disease; EU: European union; MS: Member states; MH: Major haemoglobinopathy; Thal: Thalassaemia syndromes; RD: Rare disease; ENERCA: European network for rare and congenital anaemias; TIF: Thalassaemia International Federation; WHO: World Health Organization.

## Competing interests

This study has been partially supported by a grant from Novartis.

## Authors’ information

The authors are all collaborating experts in the field of haemoglobinopathies. Michael Angastiniotis and Androulla Eleftheriou are medical advisor and executive director, respectively, of the Thalassaemia International Federation and members of the ENERCA project. Beatrice Gulbis (department of clinical Chemistry at Erasmus Hospital, Université Libre de Bruxelles, Belgium), Patricia Aguilar Martinez (CHU de Montpellier,Saint Eloi Hospital, France), Maria del Mar Mañú Pereira and Joan-Lluis Vives-Corrons (Hospital Clinic at Universitat de Barcelona) are members of the executive committee of the ENERCA project.

## Supplementary Material

Additional file 1 **Annex I: Structured questionnaire.** Annex II: Methodology. Annex III: Consulted MS organisations. Click here for file
