# Supplementary material for: Haemoglobinopathies in Europe: health & migration policy perspectives
Source: Orphanet J Rare Dis. 2014 Jul 1;9:97. doi: 10.1186/1750-1172-9-97 (PMC4094755; doi:10.1186/1750-1172-9-97)
Supplement: Additional file 1 — Annex I: Structured questionnaire. Annex II: Methodology. Annex III: Consulted MS organisations. [file 1750-1172-9-97-S1.doc]

**Annex I Structured questionnaire**

**Key data**

Data should be split between thalassaemia and SCD as possible.

- What is the number of patients with haemoglobinopathies in your country? (indicate year of estimation) How many new patients are diagnosed every year? (indicate year of estimation)
- What percentage of the overall country population do they represent?
- Is there any significant current or future trend? (e.g. increase/decrease in prevalence) And if so, what is the explanation of this trend?
- Is data on number of patients, care and sequels gathered and analysed consistently across the country? How is this done? Are there registries in your country gathering epidemiological data? How are they organised / managed? Is data analysed comprehensively to evaluate policy impact and trends and inform policy-making? Which is the responsible body to handle this process
  - Are there particular prevalence factors present in your country (e.g.: ethnicity/country of origin)
  - What are the main sequels/morbidity linked to haemoglobinopathies in your country?
  - What is the life expectancy of haemoglobinopathies patients in your country?
  - Is there data of related mortality?
- What is the estimated cost related to haemoglobinopathies? (if there is data available) –

**Policy focus**

- What is the current policy focus in the areas of health and migration in your country?
- Are there specific policies to address migrant health?
- Are there specific policies to address haemoglobinopathies specifically? In particular, has there been any measure to follow up on the WHO recommendations to establish comprehensive, integrated plans on the prevention and management of haemoglobinopathies such as thalassaemia and sickle-cell disease?[[1]](#footnote-2)
- If yes:
  - When was this plan published? What period does it cover?
  - Which body (ies) is(are) responsible?
  - What are the main components / areas addressed? Relevant areas to check:
    - Surveillance (data registries and evaluation)
    - Dissemination of information, awareness raising
    - Screening
    - Strengthening of medical services
      - To ensure holistic care
      - To improve care at primary care level
      - To improve medical genetics care
    - Healthcare professional training, in particular if your country is considered a high prevalence area
    - Awareness raising, community education
    - Active involvement of parent/patient associations, other civil organisations, local authorities
    - Support to basic and applied research on haemoglobinopathies
    - Collaboration at international level
  - Is the plan taking into account and addressing the specific socioeconomic, health and cultural contexts in your country?
  - Is this policy framework being implemented and enforced effectively in practice? Is there available data on outcomes/impact? (Or are there plans to gather this data in the future and/or regularly?)
  - How frequently is the plan reviewed/updated? What determines these updates?
- If no specific plans:
  - What are the relevant government policies/measures affecting haemoglobinopathies patients and migrant health? Are these patients dealt with in the context of other national healthcare priorities? (E.g.: cancer, CVD programmes, nephrology, migrant health programmes if any)
- Are haemoglobinopathies considered a ‘rare disease’ in your country? Are they covered by broader policy measures focused on rare diseases?
- Is there a rare disease plan in your country or is your country planning to develop one? Will haemoglobinopathies be covered by the future national plans on rare diseases and how? (National plans due by end of 2013) – how are preparatory measures being organised in order to develop the plan, how priorities and content are being decided, current status of the work, integration of this work with any specific plans on haemoglobinopathies if they exist.
- Is there a structured dialogue with stakeholders? Is there a dedicated government/stakeholder working group or forum responsible for the preparation of policy developments or implementing specific measures for rare diseases, migrant health and/or haemoglobinopathies? Are parent/patient communities involved in any other way and how?
- What are the perceived major health risks/sequels for haemoglobinopathies patients?
- Which percentage of the national healthcare budget goes to screening, diagnoses and care of haemoglobinopathies patients and/or migrant health?
- In general, are policies to address rare diseases, haemoglobinopathies, and migrant health influenced by any other country or the EU? In particular, do you think that policy developments at EU level would have a positive impact on the development and improvement of the policies aimed to ensure quality healthcare to haemoglobinopathies patients? What specific developments would be needed?
- Is international cooperation promoted in your country? In which main areas is there international cooperation to improve the care of haemoglobinopathies?

**Screening & Diagnosis**

- Are there any existing or planned policies/measures/protocols/government or professional guidelines on the screening and diagnosis of haemoglobinopathies? Which bodies are responsible? E.g. government guidelines, professional guidelines
- Is access to innovative screening techniques available? We refer to both antenatal and neonatal screening and screening of adverse effects (secondary health conditions derived from haemoglobinopathies)
- Does screening programmes include screening and diagnosis of other health sequels linked to haemoglobinopathies? (e.g. in children with SCD, Trans cranial Doppler Scanning to prevent strokes; in thalassaemia and NTDT, magnetic resonance with T2* software to detect CV and liver sequels
- Are there special screening programmes targeted at population groups at risk (e.g. migrant population; patients with relevant genetic conditions)? If yes, how are these measures organised?
- Are there targeted education/information campaigns to support screening programmes? Are they targeted at the general population and/or population groups at risk? Who organises these campaigns (e.g. government; patient organisations, etc?) Are they considered to be effective and well resourced?

**Care & Treatment**

Care organisation

- How is care to haemoglobinopathies patients organised in your country? (E.g. are these patients derived to specialised doctors, reference centres, or is there the capacity to deal with these patients at the primary care level?
- Are there current/future guidelines/protocols for care and treatment of haemoglobinopathies in order to ensure comprehensive and high quality care? Do these guidelines address the care and treatment of linked health conditions in order to ensure a holistic approach?
- Are there regular checkups/follow up organised and how? By primary care or specialised services?
- Is access to innovative care and treatment methods considered appropriate?:
- What is current level of government health spending on care and treatment of haemoglobinopathies? Is there any planned increase/decrease in this spending?

Quality care

- Is the quality of care consistent across the country?
- Are there minimum quality standards and/or minimum human and equipment resources established? If yes, which is the body responsible for setting up these standards?
- Is there specialised professional training, and/or targeted training programmes for professionals in high prevalence geographical areas?
- Is basic and applied research supported positively by policies?

Awareness and perception

- What are the main challenges/gaps perceived by patients and healthcare professionals?
- Are patients educated about care, treatment and potential health sequels and linked conditions?
- Are health professionals generally aware of issues related to haemoglobinopathies ?
- To what extent are policy stakeholders and the general public aware of the social impacts of haemoglobinopathies; impacts such as risk of stigmatisation, social exclusion, discrimination for insurance subscription (health insurance, travel insurance, mortgage), limitations in terms of social life, education and professional development of patients?
- To what degree are the national policies/measures addressing these social challenges?
- Are there education measures aimed to prevent patient stigmatisation?
- What are the main measures that should be taken to improve current care policies for haemoglobinopathies patients? / To improve health amongst the population at risk of haemoglobinopathies (e.g. migrant population).

**Policy outlook**

- Are there any policy changes in the areas of haemoglobinopathies, rare diseases or migrant health expected to occur over the next years? What? What is the expected timing?
- What changes would lead to an improvement of screening, treatment and care of haemoglobinopathies in your country?
  - In health policies?
  - In social, migration policies?
  - At national level?
  - At European level?

**Annex II : Methodology**

- Desktop research was focused on gathering information on the current policy status, ongoing and upcoming policy initiatives relevant to haemoglobin disorders, health and migration.
- Sources included national media, institutional and stakeholder (healthcare professional, patient organisations) websites.
- Individual interviews were held on by phone .
- Interviews were done to selected national stakeholders who were considered to have relevant knowledge and active involvement in the policy debate on healthcare delivery to haemoglobinopathies patients, health and migration. Activity on haemoglobinopathy and/or migrant issues. For each country, 3-4 interviews were done amongst relevant government officials/policy makers, patient groups, health professionals, migrant or health organizations **(See Annex III)**
- Interviews had a double purpose:
  - gathering information
  - gathering assessment/opinion

Both the desktop research and the individual interviews were guided by a predefined, structured questionnaire.

**Annex III: Consulted MS organisations**

| **Country** | **Organisation** |
| --- | --- |
| **Belgium** | Institut National d’Assurance Maladie-Invalidité (INAMI) |
|  | Hôpital Erasme (Brussels) |
|  | Action Drépanocytose |
| **Cyprus** | Thalassaemia International Federation |
|  | Cyprus Thalassaemia Centre |
|  | Pancyprian Thalassaemia Association |
| **France** | Centre de Compétence des maladies rares du globule rouge, Laboratoire d’Hématologie, CHU de Montpellier |
|  | Association Pour l’Information et la Prévention de la Drépanocytose (A.P.I.P.D.) |
|  | Ministère des Affaires Sociales et de la Santé |
| **Germany** | University Hospital Ulm |
|  | Universitätsklinikum Hamburg-Eppendorf |
|  | Help for Thalassemia without borders |
| **Greece** | Central Health Committee for Thalassemia |
|  | Laiko General Hospital (Athens) |
|  | Greek Thalassemia Federation (EOTHA) |
| **Italy** | Centro di Coordinamento per le Malattie Rare (Regione Lombardia) |
|  | Universitá di Verona |
|  | Associazione Talassemici e Drepanociti Lombardi (ATDL) |
| **Netherlands** | OSCAR |
|  | Paediatric Haematologist, Amsterdam Medical Centre |
|  | Erasmus Medical Centre (Rotterdam) |
|  | Ministry of Health |
| **UK** | UK Thalassaemia Society (UKTS) |
|  | Midlands & East Specialised Commissioning Group |
|  | Department of Haematology, University College London |
| **Spain** | Red Cell Pathology Unit. Hospital Clinic. University of Barcelona |
|  | Asociación Española de Lucha contra las Hemoglobinopatias y Talasemias (ALHETA) |
|  | Instituto de Investigación en Enfermedades Raras, Instituto de Salud Carlos III |
|  |  |
| **Sweden** | Akademiska Sjukhuset (Uppsala) |
|  | Södersjukhuset (Stockholm) |
|  | Ministry of Social Affairs |

1. WHO 118th Session Agenda Item 5.2, May 2006, Thalassaemia and other Haemoglobinopathies, 59th WHA, agenda Item 11.4, WHO59.20, May 2006, Sickle-Cell Anaemia. [↑](#footnote-ref-2)
